# Role of Copper in the Onset of Alzheimer’s Disease Compared to Other Metals

**DOI:** 10.3389/fnagi.2017.00446

**Published:** 2018-01-23

**Authors:** Soghra Bagheri, Rosanna Squitti, Thomas Haertlé, Mariacristina Siotto, Ali A. Saboury

**Affiliations:** ^1^Medical Biology Research Center, Kermanshah University of Medical Sciences, Kermanshah, Iran; ^2^Molecular Markers Laboratory, IRCCS Istituto Centro San Giovanni di Dio-Fatebenefratelli, Brescia, Italy; ^3^Institute of Biochemistry and Biophysics, University of Tehran, Tehran, Iran; ^4^UR 1268 Biopolymères Interactions Assemblages, Institut National de la Recherche Agronomique, Equipe Fonctions et Interactions des Protéines, Nantes, France; ^5^Department of Animal Nutrition and Feed Management, Poznan University of Life Sciences, Poznań, Poland; ^6^Fondazione Don Carlo Gnocchi Onlus, Milan, Italy

**Keywords:** Alzheimer’s disease, copper, zinc, neurodegenerative disorder, amyloid plaques, cholesterol, calcium

## Abstract

Alzheimer’s disease (AD) is a neurodegenerative disorder that is characterized by amyloid plaques in patients’ brain tissue. The plaques are mainly made of β-amyloid peptides and trace elements including Zn^2+^, Cu^2+^, and Fe^2+^. Some studies have shown that AD can be considered a type of metal dyshomeostasis. Among metal ions involved in plaques, numerous studies have focused on copper ions, which seem to be one of the main cationic elements in plaque formation. The involvement of copper in AD is controversial, as some studies show a copper deficiency in AD, and consequently a need to enhance copper levels, while other data point to copper overload and therefore a need to reduce copper levels. In this paper, the role of copper ions in AD and some contradictory reports are reviewed and discussed.

## Introduction

Alzheimer’s disease (AD) is a progressive neurodegenerative disorder described in 1907 by Alois Alzheimer. He observed amyloid plaques and neurofibrillary tangles (NFTs) in the brain of patients showing signs of dementia (Sarell, 2010). Today, AD is the most prevalent neurodegenerative disease affecting 10% of people aged 65+ and 50% of people aged 80+ (Zhang et al., 2011). In 2016, it was estimated that there were about 47 million AD patients in the world and this number is expected to increase to more than 130 million by 2050. The World Alzheimer Report evaluated the annual social and economic cost of dementia to be US$ 818 billion worldwide in 2015 and this amount is expected to increase to one trillion by 2018 (Unzeta et al., 2016).

Alzheimer’s disease is ultimately lethal, characterized by the developing damage of neuronal tissues in the brain. Signs include memory loss, paranoia, loss of reasoning powers and confusion (Sarell, 2010). Unfortunately, AD is recognized only after the manifestation of cognitive signs, which may be too late for effective treatment (Leskovjan et al., 2011). Moreover, approved drugs have inconsiderable effects on patients’ well-being, which may be because many factors are responsible for AD (Conte-Daban et al., 2016; Pickart et al., 2017). Indeed, there are several theories to demonstrate the cause of AD supported by empirical data, which have been reviewed by Armstrong (2013).

In brain regions affected in AD, such as the cortex and hippocampus, extracellular senile plaques, and intracellular NFTs accumulate (Zhang et al., 2011). Senile plaques or amyloid plaques, as the name implies, are mainly composed of small peptides called β-amyloid (Masters et al., 1985). The latter is produced from β-amyloid precursor protein (APP) through successive cleavages first by β-secretase at residue 671 and then by γ-secretase at residues 711 or 713 (residue numbering according to the APP_770_ isoform) in amyloidogenic pathway. Alternatively, APP molecules can be cleaved by α-secretase within the β-amyloid domain at residue 687 and prevent β-amyloid production in non-amyloidogenic pathway (Selkoe, 2001; Zhang et al., 2011). There are three main isoforms of APP including APP695, APP751, and APP770 with APP695 being expressed at high levels in brain compared to other isoforms (Nalivaeva and Turner, 2013). Furthermore, NFTs, mainly composed of tau proteins, is the other important factor that accumulate in AD brain (Buée et al., 2000). Tau proteins mostly expressed in neurons have the ability to induce microtubule assembly *in vitro* (Weingarten et al., 1975; Cleveland et al., 1977). In fact, microtubules, one of the main components of the cytoskeletal system, are involved in the maintenance of neuronal morphology and the formation of axonal and dendritic processes (Gendron and Petrucelli, 2009). One of the first and most severely injured brain areas in AD is the hippocampus, which is associated with neurogenesis and long-term memory storage. It is also thought to be more susceptible to metal disturbance than other brain areas. Another brain region that suffers from damage in AD due to plaque pathology is the cortex, associated with functions such as argumentation, feeling, and language (Leskovjan et al., 2011).

β-amyloid aggregations into senile plaques are one of the main characteristics of AD (Wan et al., 2011). A considerable co-localization of adenosine receptors and β-amyloid has been reported in senile plaques (Angulo et al., 2003). Adenosine, a purine ribonucleoside that has neuromodulatory and neuroprotective properties (Rahman, 2009), affects various important brain functions such as sleep, cognition, memory, and neurodegeneration (De Mendonça and Ribeiro, 1996; Porkka-Heiskanen, 1999; Ribeiro et al., 2002; Rahman, 2009). Adenosine is involved in numerous neurological disorders including AD (Cortés et al., 2015; Maiuolo et al., 2016). It exerts its various effects via its receptors, and thus managing its receptor agonists and antagonists significantly influences learning and memory (Ohno and Watanabe, 1996; Kopf et al., 1999; Corodimas and Tomita, 2001; Hauber and Bareiß, 2001; Pereira et al., 2002). On one hand, deamination of adenosine to inosine by adenosine deaminase (ADA) is one of the metabolic pathways for the catabolism of adenosine in the brain (Boison, 2006). On the other hand, ADA acts as an allosteric modulator of adenosine receptors (Cortés et al., 2015). Because of ADA’s involvement in different health disorders, the development of ADA inhibitors as feasible therapeutic agents has been considered in many studies (Cristalli et al., 2001; Saboury et al., 2003, 2004, 2005; Ataie et al., 2004, 2007; Terasaka et al., 2004a,b; Da Settimo et al., 2005; Ajloo et al., 2007; Ujjinamatada et al., 2008; La Motta et al., 2009; Bazl et al., 2012). Recently, ADA inhibitors have been proposed in perinatal hypoxia–ischemia brain injury treatment (Pimentel et al., 2013).

Polyvalent metal cations such as copper, zinc, and iron are found in high concentrations in senile plaques in AD patients’ brain (Smith et al., 1997; Lovell et al., 1998; Sayre et al., 2000; Suh et al., 2000; Dong et al., 2003; Miller et al., 2006). Furthermore, some studies in mouse models of AD revealed that in spite of accumulation of copper in senile plaques in the mouse models with neurodegeneration including 5 × FAD and CVN (Bourassa et al., 2013), no copper accumulation is observed in PSAPP mouse model with slight neurodegeneration (Bourassa et al., 2013; James et al., 2017). Considerable data point to dyshomeostasis of zinc and copper ions as the main factor of AD pathogenesis (Deibel et al., 1996; Lovell et al., 1998; Cherny et al., 1999, 2001; González et al., 1999; Huang et al., 1999, 2004; Sayre et al., 2000; Bayer et al., 2003; Phinney et al., 2003; Ritchie et al., 2003; Pajonk et al., 2005; Kessler et al., 2006; Ma et al., 2006; Maynard et al., 2006, 2002; Miller et al., 2006; Cater et al., 2008; Donnelly et al., 2008; Hung et al., 2009; Leskovjan et al., 2009; Hozumi et al., 2011; Mao et al., 2012; Arnal et al., 2013a,b; Pal et al., 2013; Singh et al., 2013) and indicate that copper metabolism proteins are associated with AD (Phinney et al., 2003; Southon et al., 2013; Pal et al., 2014).

Different authors have put forward various models of the toxicity of copper involvement in AD. The most accredited one proposes the gain-of-function of β-amyloid (Bush et al., 2003; Bush and Tanzi, 2008) after binding Cu^2+^ (Multhaup et al., 1996). Alternative and more recent hypotheses (Lee et al., 2005; Cavaleri, 2015; Kepp, 2016) propose a protective role of β-amyloid against an excess of toxic metals within the brain, designating β-amyloid loss-of-function as a pathogenic process in the disease (Hua et al., 2011; Kepp, 2016). APP is thought to possess a normal function in metal export from neurons, and a putative loss of the soluble, functional β-amyloid monomer could cause copper build-up in the cell (Kepp, 2016).

Scientific evidence has shown that metal ion binding to β-amyloid accelerates amyloid aggregation, which could finally damage the neurons in AD (Pithadia and Lim, 2012). The involvement of copper in AD is controversial, as some studies show copper deficiency in AD, and consequently a need to enhance copper levels (Borchardt et al., 1999; Kessler et al., 2005, 2008a,b; Exley, 2006; Jiao and Yang, 2007; Vural et al., 2010; Kaden et al., 2011; Exley et al., 2012), while other data point to copper overload and therefore a need to reduce copper levels (Cherny et al., 2001; Sparks et al., 2006; Hua et al., 2011; Luo et al., 2011; Ceccom et al., 2012; Eskici and Axelsen, 2012; Brewer, 2014; Squitti et al., 2014b; Yu et al., 2015). An aberrant copper homeostasis with an increase in the labile pool of copper and a decrease in the copper bound to protein is the main up-dated interpretation (Kepp, 2016; Squitti et al., 2016). In this paper, the role of metal ions, particularly copper, in AD is reviewed and discussed.

## Copper Ion Toxicity in AD

The key event in AD is the formation of fibrils and plaques in AD patients’ brain. Plaques are mainly made of β-amyloid peptide, the natural peptide that is produced in the brain and exists at nanomolar concentration levels in cerebrospinal fluid (CSF) and serum (Masters et al., 1985; Vigo-Pelfrey et al., 1993). On the other hand, a high concentration of trace metals, including copper, is observed in amyloid plaques (Miller et al., 2006). Interestingly, some data show that copper distribution in the brain does not correspond to β-amyloid plaques distribution in TASTPM mice model (Torres et al., 2016) with plaque pathology but not appreciable neuronal loss (Howlett et al., 2004). Copper is a necessary trace metal in nervous system development since disruption of its homeostasis leads to neurodegenerative disorders like Menkes and Wilson’s diseases (Waggoner et al., 1999). Cu^2+^ ions bind to β-amyloid peptides with high affinity (Atwood et al., 2000; Sarell et al., 2009; Barritt and Viles, 2015; Mital et al., 2015; Drew, 2017) and increase the proportions of β-sheet and α-helix structures in amyloid peptides, which can be responsible for β-amyloid aggregation (Dai et al., 2006). Various concentrations of Cu^2+^ ions enhance fibril formation while binding of copper ions to β-amyloid noticeably increases its toxicity for cells (Dai et al., 2006; Sarell et al., 2010). In addition, substoichiometric concentrations of Cu^2+^ are more toxic to cells (Sarell et al., 2010).

Fibril formation is highly pH-dependent and Cu^2+^ ions cause it to occur at physiological pH. However, the formation of amorphous aggregates dominates in acidic conditions (Jun et al., 2009; Sarell, 2010; Lv et al., 2013). In a proton-rich environment, β-amyloid (Aβ_40_) possesses two copper binding sites, and its second bound Cu^2+^ion causes the formation of amorphous aggregates by preventing the conformational transition of β-amyloid into amyloid fibrils (Jun et al., 2009).

The production of Reactive Oxygen Species (ROS) is a key factor in β-amyloid toxicity toward neurons, which is dependent on metal ion redox properties. Copper ions in complex with β-amyloid fibrils produce hydrogen peroxide, in the presence of biological reducing agents (Parthasarathy et al., 2014). When the ratio of copper to peptide increases, hydrogen peroxide levels and the production of hydroxyl radicals increase, and the morphology of aggregates changes from fibrillar to amorphous (Mayes et al., 2014). Although previous studies have presented ROS as fatal molecules provoking neurodegeneration, the accumulated evidence shows that some ROS act as essential molecules in processes underlying cognition and memory formation (Klann, 1998; Yermolaieva et al., 2000; Knapp and Klann, 2002a,b; Kamsler and Segal, 2003, 2004; Hu et al., 2006; Kishida and Klann, 2007). On the other hand, some results imply that the copper-amyloid complex produces fewer ROS than free copper ions (Nakamura et al., 2007). According to *in vitro* data, oligomeric and fibrillar forms of β-amyloid inhibit H_2_O_2_ generation at higher concentrations of Cu^2+^. In addition, the fibrillar form generates less H_2_O_2_ than the oligomeric form (Fang et al., 2010).

Copper toxicity in AD brains is attributed to the oxidized form of copper ions, i.e., Cu^2+^, (Brewer, 2015; Greenough et al., 2016). In contrast, other data show that copper ions are only transported in their reduced form, i.e., Cu^1+^, (Macreadie, 2008). Some studies suggest that Cu^2+^ bypasses the liver (Brewer, 2015). Otherwise, some data show that the removal of Cu^1+^ from β-amyloid, hinders the formation of oligomers and prevents ROS production (Atrián-Blasco et al., 2015). A study of the affinity of the soluble copper-binding domain of the β-amyloid peptide for Cu^1+^shows that it binds to β-amyloid stronger than Cu^2+^ suggesting Cu^1+^ is the relevant *in vivo* oxidation state (Feaga et al., 2011). Both Cu^1+^ and Cu^2+^ inhibit β-amyloid degradation by insulin-degrading enzyme, but Cu^1+^ cations act as irreversible inhibitors (Grasso et al., 2011). Copper ion by reduction from Cu^2+^ to Cu^1+^ protects proteins against free radicals (Deloncle and Guillard, 2014).

A meta-analysis (Schrag et al., 2011) and subsequent studies (James et al., 2012; Szabo et al., 2016) demonstrated that the concentration of total copper is decreased in the brain of AD patients, while the concentration of labile copper is increased in the most affected regions of the AD brain (James et al., 2012). In addition, AD cortical tissues (James et al., 2012) and the cortex of mice with Traumatic Brain Injury show an elevated binding capacity for Cu^2+^ (Peng et al., 2015). Another study shows that in APP^sw/0^ mouse model of AD, which shows parenchymal plaques but no neuronal loss (Elder et al., 2010; Sasaguri et al., 2017), unlike in the control mouse in which the metal accumulates in the capillaries, copper ions accumulate in brain parenchyma. These ions could bind to β-amyloid and stimulate β-sheet conformation, aggregation, and toxicity (Singh et al., 2013).

**FIGURE 1:**
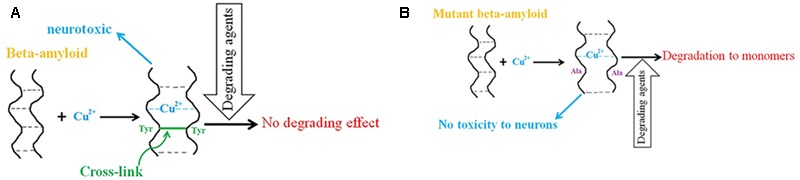
The role of copper in β-amyloid neurotoxicity in AD. **(A)** Copper binding to β-amyloid peptides leads to the formation of dityrosine-linked β-amyloid dimers, which resist degradation into monomers and have neurotoxic properties. **(B)** Mutant β-amyloids (Tyr to Ala) in the presence of copper ions have no toxicity effect on neurons and degrade to monomers by degrading agents.

In the “amyloid cascade hypothesis,” plaque formation is a main event in AD pathology but it is sometimes preceded by neurodegeneration, and plaque clearance by immunization of AD patients does not prevent disease progression (Chui et al., 1999; Oddo et al., 2003; Holmes et al., 2008; Bayer and Wirths, 2010; Bittner et al., 2012; Wright et al., 2013; Xie et al., 2013; Jung et al., 2015). Moreover, some studies show that senile plaques exist in cognitively normal people (Jack et al., 2010; Sperling et al., 2011; Swerdlow, 2011; Esparza et al., 2013) and, despite an equivalent plaque presence, the concentration of brain amyloid oligomers is higher in AD patients than in normal cases. The “Toxic oligomers hypothesis” explains these events by suggesting that small, diffusible oligomers are responsible for toxicity, and not the amyloid plaques (Naylor et al., 2008; Sarell, 2010). The oligomers derived from cell culture have unusually high chemical stability and resist degradation into monomers by various degrading agents, supporting the existence of covalent cross-links between the oligomers (Podlisny et al., 1995; Walsh et al., 2002; Lesné et al., 2006; Naylor et al., 2008). Based on *in vitro* experiments, Cu^2+^ binding to β-amyloid can lead to the formation of dityrosine-linked dimers of β-amyloids found in AD (Atwood et al., 2004; Haeffner et al., 2005; Bush and Tanzi, 2008; Streltsov et al., 2008; Al-Hilaly et al., 2013). In the presence of Cu^2+^, the dimer conformation changes from parallel to anti-parallel and is stabilized by the occupied copper binding sites (Hane et al., 2013). However, the same authors showed later that Cu^2+^ at nanomolar concentrations has no effect on peptide–peptide affinity in the amyloid dimer (Hane et al., 2016). Other authors have demonstrated that binding of Cu^2+^ ions induces structural changes in the amyloid dimer resulting in N-termini interactions within it (Lv et al., 2013). The mutant dimer that is unable to produce cross-links provides supporting evidence for the toxicity of Cu^2+^cross-linked dimers because the mutant dimer’s properties are the same as those of the wild type dimer except that it has no neurotoxicity (Barnham, 2004; **Figure 1**). In addition, other studies suggest that the toxicity of the cross-linked dimer is due to enhanced membrane binding (Ciccotosto et al., 2004).

Elimination of Cu^2+^ from β-amyloid prevents amyloid aggregation *in vitro* (Wu et al., 2008; Behbehani et al., 2012), and promotes β-amyloid degradation, and prevents H_2_O_2_ formation. Hence, it also decreases cell mortality (Wu et al., 2008). Because of these positive effects of copper elimination, some studies have targeted copper chelators as suitable drugs (Moret et al., 2006; Geng et al., 2012; Nguyen et al., 2014; Savelieff et al., 2014; Hauser-Davis et al., 2015; Hung et al., 2015; Yang et al., 2016). However, the most recently published review on copper chelation therapy states that the results in human clinical trials are discouraging (Drew, 2017), even though some authors have refuted this interpretation (Squitti et al., 2017a). Furthermore, studies in Tg2576 mouse model of AD, which shows parenchymal plaques but no neuronal loss (Elder et al., 2010; Sasaguri et al., 2017), show that although the use of chelator helps to prevent AD, it is inefficient in AD treatment suggesting that systemic copper removal is useful only in the early stages of the disease (Quinn et al., 2010). Interestingly, there is some evidence that making changes in brain copper uptake in the primary stages can have a considerable effect on amyloid pathology (Lang et al., 2013).

Until 2012, a number of ambiguous results published previously fueled a debate about copper levels in AD patients. Overall, six meta-analyses have been carried out in the last 6 years to evaluate copper concentrations in AD in different biological matrices (serum, plasma, and cerebrospinal fluid). These meta-analyses, combining data collected from studies published between 1984 and 2017 (Bucossi et al., 2011; Ventriglia et al., 2012; Schrag et al., 2013; Squitti et al., 2014a; Wang et al., 2015; Li et al., 2017), provide unequivocal results: total copper (Bucossi et al., 2011; Ventriglia et al., 2012; Schrag et al., 2013; Squitti et al., 2014a; Wang et al., 2015; Li et al., 2017) and “free” copper (Squitti et al., 2014a) are higher in the serum–plasma of AD patients in comparison with healthy controls. More specifically, the large stand most recent meta-analysis (total pool of subjects analyzed: 2128 AD vs. 2889 healthy controls) includes a total of 35 studies: 18 report an increase, 14 no difference, and one a decrease in values of copper in the serum–plasma in AD compared to healthy controls (Li et al., 2017). Three additional studies appeared after the publication of this consensus result (Guan et al., 2017; Pu et al., 2017; Talwar et al., 2017), reporting increased concentrations of Cu^2+^ in AD patients vs. controls.

Recent studies have contributed to unraveling further the initial controversy, demonstrating that the increased concentration of serum copper in AD can be explained by the increased concentrations of the plasma fraction of the “free” copper pool in the blood, which is detected in only 50–60% of AD patients (Squitti et al., 2016; Szabo et al., 2016; Tecchio et al., 2016; Talwar et al., 2017). An older study also indicated that serum copper concentration rises in a special type of AD (González et al., 1999). Some studies have proposed a genetic basis for this AD subtype as an explanation of this observation (González et al., 1999; Liu et al., 2013; Squitti et al., 2013, 2017b; Mercer et al., 2017).

## Brain Copper Deficiency in AD

A significant reduction in copper ion levels is observed in the hippocampus and amygdale areas of AD patients compared to age-matched control subjects (Deibel et al., 1996). In addition, a reduction in net copper is found in the brain of TgCRND8 AD mice model (Phinney et al., 2003), and this model exhibits parenchymal amyloid deposition but no neuronal loss (Sasaguri et al., 2017). As mentioned above, a meta-analysis indicates that copper is significantly decreased in the brain of AD patients (Schrag et al., 2011). An analysis of the human brains of deceased patients with dementia concludes that defective regions have a very low copper content (Pickart et al., 2017). The copper content of aged human brains has a significant negative correlation with the degree of severity of amyloid plaques (Exley et al., 2012). Based on the results showing a significant reduction in copper ion in AD patients compared to controls (Giacoppo et al., 2014), it has been hypothesized that AD is a result of copper deficiency (Klevay, 2008). An alternative, and more comprehensive, interpretation, which can explain the copper quantification results of meta-analyses in serum–plasma and the meta-analysis in the brain, is that the copper decrease in the brain is a sign of an aberrant copper homeostasis, which resembles Wilson’s disease (Fujiwara et al., 2006). Interestingly, some data indicate that copper deficiency in AD patients is independent of their diet (Giacoppo et al., 2014).

In the presence and absence of copper, APP molecules are cleaved in non-amyloidogenic and amyloidogenic pathways, respectively. The latter pathway results in amyloid production. Some data show that copper ions inhibit amyloid production by interacting with a γ-secretase complex (Gerber et al., 2017) or by affecting APP dimerization (Kong et al., 2008). Otherwise, copper ions enhance APP exposure on the cell surface by both increasing its exocytosis and decreasing its endocytosis (Acevedo et al., 2011). Cu deficiency, as observed in AD patients, enhances β-amyloid production and accumulation by inducing the amyloidogenic processing of APP (Bayer et al., 2003). However, its molecular mechanism is still unclear (Wild et al., 2017).

The mutations in genes encoding proteins required for copper ion uptake in mammalian systems lead to early-onset familial AD (Southon et al., 2013). On the other hand, enhancement of intracellular copper levels through addition of dietary copper in APP/PS1 transgenic AD mice (Crouch et al., 2009) and APP23 transgenic mice (Bayer et al., 2003) with parenchymal plaques but no neuronal loss (Elder et al., 2010; Sasaguri et al., 2017), causes a reduction in AD pathology. Based on the report of low brain copper in several neurodegenerative disorders, impairment in copper protection against free radicals has been proposed as the main cause of these disorders (Deloncle and Guillard, 2014).

## Copper and Zinc Ions in AD

A significant elevation in Zn^2+^ is found in the AD hippocampus and amygdale area (Deibel et al., 1996), and in the AD neuropil compared to controls (Lovell et al., 1998), although some data indicate an elevation in Zn^2+^ content in the AD brain cortex (Religa et al., 2006). Accordingly, a meta-analysis revealed no significant changes in zinc content in the AD neocortex (Schrag et al., 2011). Nevertheless, three meta-analyses (Ventriglia et al., 2015; Wang et al., 2015; Li et al., 2017) were recently carried out on plasma and serum zinc levels in AD patients and healthy controls. One study (Ventriglia et al., 2015) (the pooled sample size included 777 AD vs. 1728 healthy controls) analyzed a total of 16 studies and concludes that AD patients show a decrease in serum zinc levels compared to healthy controls. Ventriglia et al.’ (2015) study (the pooled sample size included 287 AD vs. 166 healthy controls) analyzed a total of five studies on plasma zinc: two indicate a decrease and three no significant differences in plasma zinc in AD patients compared to controls. The other study (Wang et al., 2015) (the pool of subjects included 862 AD patients vs. 1705 controls) analyzed a total of 17 studies: 10 indicate a decrease, and seven no significant differences in serum zinc in AD patients compared to controls. The most recent meta-analysis by Li et al. (2017) analyzed 22 studies with a total pool of 1027 patients with AD and 1949 healthy controls: three studies indicate an increase, 18 studies a decrease, and one study no differences in serum zinc between AD and healthy controls. Interestingly, an older study found that a rise in serum Zn^2+^ occurs in a special type of AD (González et al., 1999). As the absence of fibrinogen in serum is the main difference between serum and plasma compositions and fibrinogen-β-amyloid interactions are involved in AD progression (Cortes-Canteli et al., 2012; Derakhshankhah et al., 2016), it is likely that the reduction in serum Zn^2+^ in AD patients is due to its interactions with fibrinogen. The rise in zinc levels is accompanied by a rise in tissue amyloid levels (Religa et al., 2006). AD brain tissue contains hot spots of metal ions especially enriched by copper and zinc ions. In 2006, it was reported for the first time that β-amyloid plaques and hot spots of accumulated metal ions co-localize (Miller et al., 2006).

**FIGURE 2:**
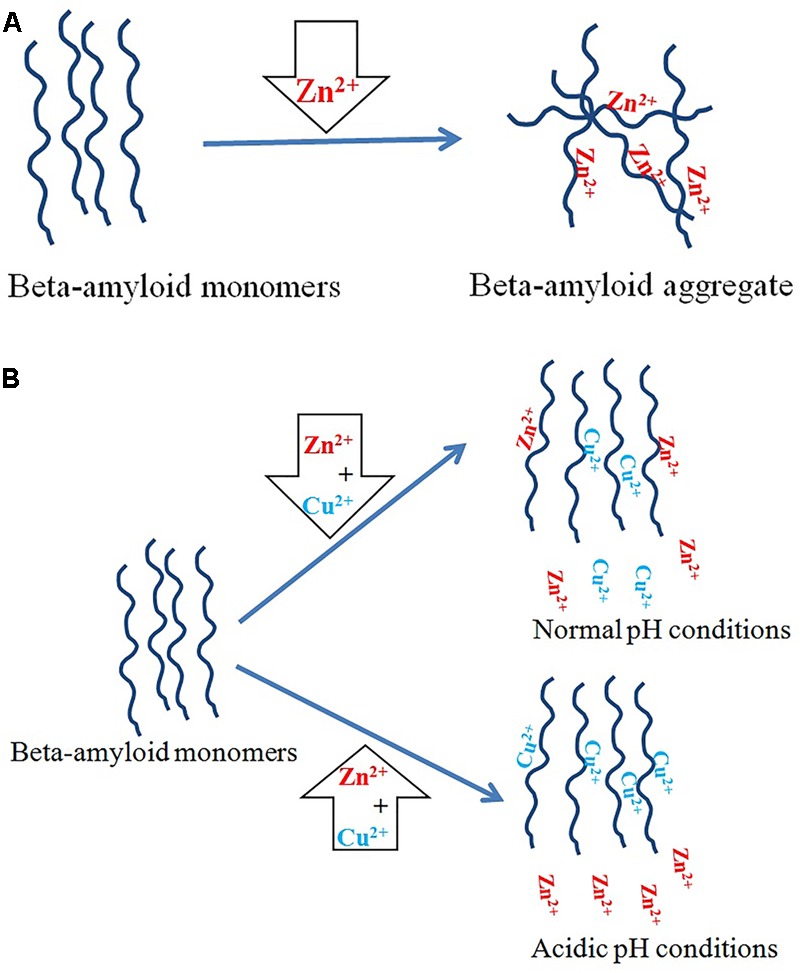
Copper and zinc ions in AD. **(A)** Zinc ions promote β-amyloid aggregation. **(B)** Copper ions have an inhibitory effect on aggregation induced by zinc ions. Under physiological conditions, β-amyloid binds the same ratio of copper and zinc ions, but in acidic pH copper ions replace zinc ions overall.

It has been suggested that zinc ions induce β-amyloid aggregation *in vitro* whereas Cu^2+^ ions inhibit it through competing with zinc for histidine residues. The strongest inhibitory effect occurs at a copper: β-amyloid molar ratio of about four. Above this value, copper ions themselves induce aggregation (Suzuki et al., 2001). Interestingly, previous studies have shown that β-amyloid binds to three or four Cu^2+^ ions at pH 7.0 (Atwood et al., 1998). Studies on synthetic Aβ(1–40) and Aβ(1–42) peptides show that at physiological pH, β-amyloid binds the same ratio of copper and zinc ions, whereas in acidic conditions copper ions replace zinc ions (**Figure 2**; Atwood et al., 2000; Roberts et al., 2012). Cu^2+^ and Zn^2+^ ions inhibit β-amyloid fibrillization, promoting instead the formation of non-fibrillar aggregates *in vitro*. In addition, zinc ions have a threefold stronger inhibitory effect than copper ions (Tõugu et al., 2009). Both Cu^2+^ and Zn^2^ ions prevent the formation of soluble fibrils if incubated with Aβ(1–42) *in vitro* (Bolognin et al., 2011). Some authors have also reported that small, soluble oligomers play the main role in β-amyloid neurotoxicity (Tabner et al., 2011). Cu^2+^ ions form stable and soluble 1:1 complexes with β-amyloid (Aβ_40_) while Zn^2+^ ions cause their partial aggregation (Tõugu et al., 2008). Cu^2+^-induced aggregates are toxic to neurons only in the presence of ascorbate, while monomers and zinc-induced aggregates are not toxic (Tõugu et al., 2009).

The role of zinc supplementation in AD remains controversial according to a systematic review in 2012 reporting that Zn^2+^ in the diet has no effect on cognitive decline (Loef et al., 2012) even though the Zenith and the Zincage studies suggested some effects (Simpson et al., 2005; Marcellini et al., 2006; Maylor et al., 2006). However, 3xTg-AD mice model with background neuropathology similar to AD patients (Sasaguri et al., 2017) displayed a delay in memory impairment (Corona et al., 2010). Furthermore, Tg2576, TgCRND8 and CRND8/E4 models exhibited a potentiationin memory impairment (Linkous et al., 2009; Railey et al., 2011; Flinn et al., 2014). Moreover, Tg2576 mice and Sprague Dawley rats showed a lower brain copper and amyloid burden (Harris et al., 2014), and a higher cognitive performance, respectively (Sandusky-Beltran et al., 2017).

Selective release of Cu^2+^ and Zn^2+^ ions inside the cells by complexes can decrease the level of β-amyloids (Donnelly et al., 2008). B-amyloid peptides in the presence of physiological concentrations of copper and zinc ions are degraded but at higher concentrations of these cations they aggregate (Strozyk et al., 2009). In agreement with this finding, the use of a copper–zinc chelator rapidly causes a considerable decrease in the deposition of brain amyloid in APP2576 transgenic mice (Cherny et al., 2001). Other authors suggest that the addition of chelators to β-amyloid aggregates induces their rapid fibrillization *in vitro*. In addition, a long incubation of non-fibrillar aggregates transforms them into fibrillar forms (Tõugu et al., 2009). These findings confirm that the fibrillar form of β-amyloid is its most stable state (Tõugu et al., 2009; Breydo et al., 2016). Zn^2+^ and Cu^2+^ ions induce refolding of β-sheets into α-helix structures leading to oligomerization and membrane penetration of amyloid peptides (Curtain et al., 2001). Copper and zinc ions both induce a β-amyloid conformational change but zinc has a greater effect (Yao et al., 2012).

## Iron Ions in AD

Iron is a redox active metal and its brain level varies according to specific regions of the brain. Commonly, the brain areas responsible for motor functions are found to have higher iron concentrations than non-motor related areas (Kozlowski et al., 2012). Iron also has a high concentration at the periphery of amyloid plaques (Greenough et al., 2013). Nevertheless, there is controversy about the level of iron outside plaques in the AD brain (Schrag et al., 2011). Contrary to copper and zinc, very few structural studies have been reported on iron coordination to β-amyloid (Hureau, 2012). While copper and zinc co-purify with β-amyloid extracted from plaques, iron does not co-localize within β-amyloid deposits (Grundke-Iqbal et al., 1990; Quintana et al., 2006; Kozlowski et al., 2012) and *in vitro* studies have shown that β-amyloid binds to iron with low affinity (Viles, 2012; Greenough et al., 2013). After Louis Goodman’s case studies performed in the 1950s, a relationship between regions of AD pathology and iron accumulation was proposed (Everett et al., 2014). Some data show that β-amyloid reduces Fe^3+^–Fe^2+^ in solution *in vitro* (Khan et al., 2006; Everett et al., 2014). While some authors argue that the direct coordination of Fe^3+^ to β-amyloid is impossible at natural pH and that Fe^2+^ is air-sensitive and oxidizes to Fe^3+^ during measurements (Hureau, 2012), others report a potential pro-aggregating function for Fe^2+^ and Fe^3+^ (Masters and Selkoe, 2012) and some data show a higher affinity for β-amyloid to Fe^2+^ relative to transferrin (Jiang et al., 2009). Three meta-analyses have been carried out in the last 4 years to assess iron concentrations in AD in serum, cerebrospinal fluid, and brain (Tao et al., 2014; Wang et al., 2015; Li et al., 2017). Tao et al.’s (2014) meta-analysis (total pool of subjects analyzed: 1813 AD vs. 2401 healthy controls) includes a total of 43 studies; 21 consider serum iron; seven consider CSF iron and 19 investigate iron in various brain areas in AD. Their results shows that serum iron significantly decreases in AD compared to controls. CSF iron shows no difference while some specific brain areas show an increase in iron concentration. Wang et al.’s (2015) meta-analysis (total pool of subjects analyzed: 1084 AD vs. 1319 healthy controls) includes a total of 18 studies showing no difference in serum iron but, after exclusion of the study producing high heterogeneity, their results conclude that serum iron levels are significantly lower in AD subjects. Li et al.’s (2017) meta-analysis (total pool of subjects analyzed: 1379 AD vs. 1664 healthy controls) includes 25 studies that show overall no significant difference in serum iron between AD and controls but, after excluding two studies with high heterogeneity, serum iron is significantly lower in AD cases.

## Copper and Lipid Rafts in AD

There is considerable evidence that the amyloidogenic pathway takes place in lipid rafts, which are specific membrane domains enriched in cholesterol. The enzymes that cleave APP to β-amyloid peptides are found in lipid rafts (Riddell et al., 2001; Hung et al., 2009). Cellular copper deficiency results in an accumulation of copper ions in cholesterol-rich lipid rafts. In fact, the copper level in lipid rafts is inversely related to cellular copper, which results in enhanced copper-amyloid complex formation under copper deficiency conditions in AD (Cater et al., 2008; Hung et al., 2009).

Clearly, diet is a critical factor in the progression of AD. Several studies indicate the key role of fat in AD (Grant, 1997; Sparks and Schreurs, 2003; Morris et al., 2006; Brewer, 2012). The effective role of Cu^2+^ and cholesterol overload in neurodegeneration has been reported (Arnal et al., 2013b; Wong et al., 2014). In rats treated with copper and cholesterol, a significant change in visuo-spatial memory is detected (Arnal et al., 2013b) while the administration of dietary cholesterol plus copper-supplemented drinking water (in the form of copper sulfate) induces an accumulation of β-amyloid in rabbit brain (Larry, 2004). The binding of Cu^2+^ ions to β-amyloid causes oxidation of cholesterol, and the generation of H_2_O_2_ (Hung et al., 2009) and other lipid peroxidation products accumulating in AD patients’ brain (Murray et al., 2007) and in Tg2576 transgenic mouse model (Puglielli et al., 2005). In fact, previous unsuccessful therapeutic attempts and recent findings regarding β-amyloid accumulation at lipid rafts have led to a new hypothesis that neurotoxicity in AD is the result of the association of small soluble amyloid oligomers with the plasma membrane (Drolle et al., 2014; Kotler et al., 2014; Arbor et al., 2016). B-amyloid membrane binding is mediated by its interactions with phosphatidylserine (Ciccotosto et al., 2011).

A highly toxic isoform of β-amyloid that accumulates in AD patients’ brain has a great capacity to induce lipid peroxidation. It also alters the calcium influx by binding to cell membranes. This isoform does not lead to an increase in ROS but it causes instead a reduction in plasma membrane integrity and an increase in dityrosine-β-amyloid oligomers (Gunn et al., 2016). Other studies have suggested that the toxicity of the cross-linked dimer is due to enhanced membrane binding (Ciccotosto et al., 2004).

**FIGURE 3:**
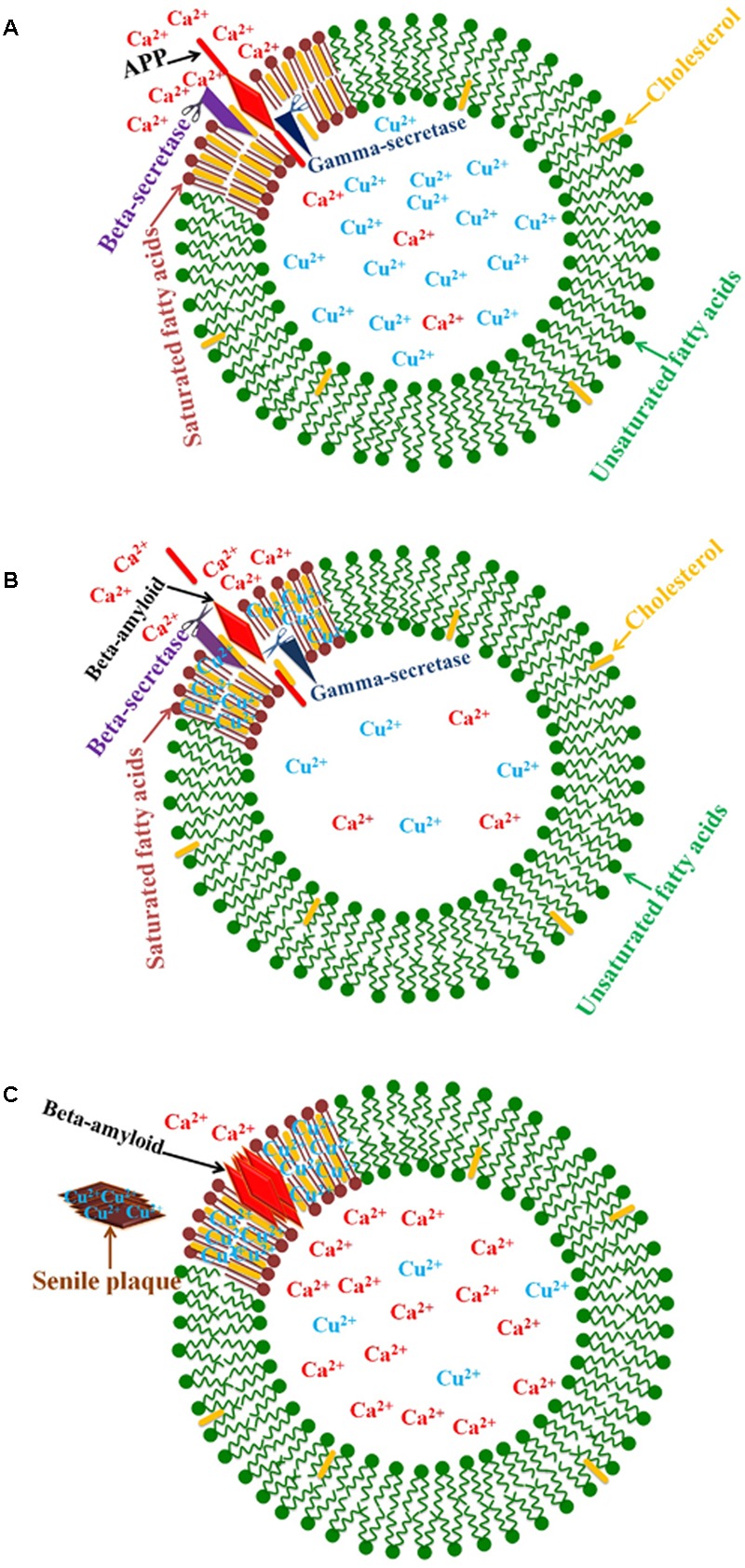
Schematic diagram of the relationship between copper and lipid rafts in AD. **(A)** The enzymes that cleave APP to β-amyloid peptides are found in lipid rafts. **(B)** Cellular copper deficiency results in copper ions accumulating in cholesterol-rich lipid rafts, and β-amyloid production increases because of greater enzyme activity due to a rising copper concentration. **(C)** β-amyloid peptides compose the oligomeric pores in membranes via the cholesterol-binding domain. These pores are calcium channels that disrupt calcium homeostasis in neural cells.

Simulation experiments show that increased cell membrane cholesterol results in some changes in the membrane; namely, an enhancement of surface hydrophobicity and a reduction in bilayer mobility. These membrane changes induce amyloid binding to the cell membrane and cause β-amyloid to refold into a helical or unstructured form. B-amyloid is stabilized on the membrane surface or inserted into the bilayer with the help of calcium ions (Yu and Zheng, 2012). In fact, β-amyloid peptides compose the oligomeric pores in the membrane via the cholesterol-binding domain (Lashuel et al., 2002; Di Scala et al., 2014). Channel formation is cholesterol-dependent and occurs in the presence of at least 30% cholesterol in lipid bilayer membranes. These pores are ion channels that disrupt calcium homeostasis in neural cells and have led to the return of the “calcium hypothesis” of AD (Di Scala et al., 2014, 2016; **Figure 3**). In the absence of copper, β-amyloids are cleared to the blood even if there are increased cholesterol levels, while in the presence of copper β-amyloid accumulate in the brain (Sparks, 2007). Otherwise, copper ions in the absence of β-amyloid are not toxic to cells (Sarell et al., 2010).

## Conclusion

Alzheimer’s disease is a progressive degenerative disease characterized by the presence of senile plaques in the AD patient’s brain. It is a multifactorial disease with a number of genetic and environmental factors likely involved. Previous studies have identified these plaques as toxic elements in AD. However, other studies have demonstrated that 20–40% of healthy people possess senile plaques in their brains (Jack et al., 2010). Moreover, cell death sometimes precedes plaque formation. A number of studies have focused on ROS generation by a copper-amyloid complex and propose ROS as toxic elements in AD. Still other authors have shown that ROS are the basis of memory formation. In addition, fibrils and oligomers at higher copper ion concentrations inhibit the production of ROS. As an alternative, the toxic oligomers hypothesis seems strong, showing that soluble oligomers interact with cell membranes, inducing in them the formation of calcium channels. Collectively, it seems that a disruption of copper control mechanisms occurs in AD, affecting the compartmentalization of the metal in different tissues and organs. Some authors have observed that the increased levels of the labile pool of copper in the brain (James et al., 2012) and in the periphery (Squitti et al., 2014a) bring about a deficiency of copper in the brain and an increase in the labile pool of copper in the brain and in “free copper” in the blood. This is the picture implied by the conditions of Wilson’s disease (Fujiwara et al., 2006). A possible interpretation is that a copper deficiency in brain cells is harmful because of amyloid production, and then copper deficiency leads to a rise in copper levels on lipid rafts. In conditions of copper deficiency, the amyloid-copper complex increases in lipid rafts because of higher levels of both copper and amyloid as well as the high affinity of amyloid peptides for copper. Copper ion binding and proximity to the cell membrane induce the refolding of amyloid peptides, and finally their oligomerization and interactions with cell membranes. The root of copper deficiency in the brain cells seems to be an important factor in AD. Since it is accompanied by copper enrichment in lipid rafts, one can argue that an elevation in lipid raft domains could lead to copper deficiency in the brain, thus targeting lipid rafts could be an effective therapeutic approach. Indeed, some data show that disrupting lipid rafts (by omega-3 fatty acids) delays the incidence of the disease (Cooper, 2003; Dannenberger et al., 2013; Arbor et al., 2016).

## Author Contributions

The review was written by SB with assistance and feedback from AS, RS, TH, and MS. All authors approved the final version of the manuscript.

## Conflict of Interest Statement

The authors declare that the research was conducted in the absence of any commercial or financial relationships that could be construed as a potential conflict of interest.
